# Sources of Error in UV Radiation Measurements

**DOI:** 10.6028/jres.106.030

**Published:** 2001-08-01

**Authors:** Thomas C. Larason, Christopher L. Cromer

**Affiliations:** National Institute of Standards and Technology, Gaithersburg, MD 20899-8441; National Institute of Standards and Technology, Boulder, CO 80303

**Keywords:** calibration, error, measurement, radiometry, ultraviolet, uncertainty

## Abstract

Increasing commercial, scientific, and technical applications involving ultraviolet (UV) radiation have led to the demand for improved understanding of the performance of instrumentation used to measure this radiation. There has been an effort by manufacturers of UV measuring devices (meters) to produce simple, optically filtered sensor systems to accomplish the varied measurement needs. We address common sources of measurement errors using these meters. The uncertainty in the calibration of the instrument depends on the response of the UV meter to the spectrum of the sources used and its similarity to the spectrum of the quantity to be measured. In addition, large errors can occur due to out-of-band, non-linear, and non-ideal geometric or spatial response of the UV meters. Finally, in many applications, how well the response of the UV meter approximates the presumed action spectrum needs to be understood for optimal use of the meters.

## 1. Introduction

The variety of applications of ultraviolet (UV) light and the consequent need for accurate UV measurements have increased enormously over the last 20 years. In some cases, the UV radiation from a source is of interest (e.g., tanning booths and solar radiation). At other times, the action or chemical reaction initiated by UV irradiation of a system is of interest (e.g., water purification, UV curing, and semiconductor photolithography). Finally, UV radiation has a cumulative deleterious effect on biological systems; there are consequently health and safety requirements for the accurate measurement of UV radiation.

**Fig. 1:**
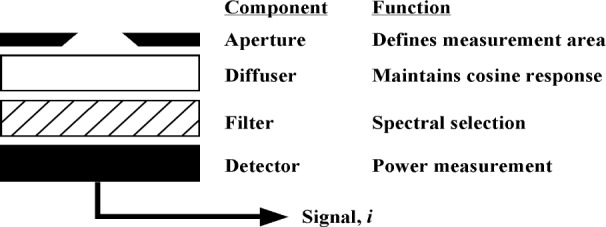
Diagram of UV radiometer with optical components highlighted.

Considerable effort has been made to produce simple instrumentation to meet these wide-ranging UV measurement needs. The typical UV meter or radiometer is composed of a number of simple optical elements, as shown in Fig. 1. The incident radiation passes through an aperture that limits the active area of the system. A diffuser is often placed after the aperture and is used to improve the angular response and spatial uniformity of the instrument. An optical filter is then employed to select the spectral region of the incident optical radiation that strikes the detector.

The signal *i* observed from such a UV radiometer is the integral of the product of the instrument responsivity *S*(*λ*) and the irradiance distribution of the source *E*(*λ*) [1]:

$$i=\int_{\lambda }E\left(\lambda \right)\cdot S\left(\lambda \right)\cdot d\left(\lambda \right).$$ (1)

The instrument responsivity is a function of the responsivity of the detector as well as the transmittance of the diffuser and optical filter.

To fully understand the accuracy of such a UV meter, the optical properties of its components and the spectral responsivity should be known as well as the relative spectral distribution of the source. Additionally, the UV meter will seldom perform ideally, and out-of-band, non-linear, and non-ideal geometric or spatial response must be characterized to achieve the lowest uncertainties. However, most UV meters are supplied from the manufacturer with a calibration at a specific wavelength, and only a nominal wavelength band is specified. In addition, the spectral distribution of the source being measured is often unknown. The purpose of this paper is to illustrate that considerable thought must be given to the utilization and calibration of these simple devices in order to understand and minimize measurement errors.

## 2. Sources of Error

It is important to define, at the outset, the physical quantity that is to be measured and the level of uncertainty needed to achieve the measurement goals. The measurement requirements for the UV meter can be very different: spectrally integrated irradiance (W/cm^2^) in the UV-A (315 nm to 400 nm) or UV-B (280 nm to 315 nm) regions as in the case of solar irradiation; a single wavelength dose or exposure (J/cm^2^) as in the case of semiconductor photolithography; or an effective or weighted dose (Effective J/cm^2^) as in the case of biological action spectra.

The sources of error in optical radiation measurements described here are not new to radiometry. These errors in addition to measurement techniques and procedures are well documented in the field of photometry. However, these topics are less well known in the UV radiation measurement community, especially among novice users of UV measurement instruments.

Due in part to increasing UV applications, recent publications specifically address UV meter calibration and characterization [2, 3]. In the following, we discuss common sources of error in UV radiation measurements, including out-of-band contributions to the signal, non-ideal geometric properties (non-ideal cosine response in the meters), and poor matching to a defined action spectrum.

Other sources of error have been discussed in the literature and will not be discussed here. These include environmental factors such as temperature and humidity, which can lead to wavelength-dependent responsivity changes in UV meters. In addition, UV radiation itself induces aging of the optical elements of meters.

Finally, optical detectors used in UV meters have a finite range over which they have an output signal linearly proportional to the incident irradiance. UV meters should be tested to verify that they are in the linear range both for the irradiance level used in practice as well as for the smaller levels typically used for calibration.

## 3. Out-of-Band/Non-Ideal Responsivity

**Fig. 2:**
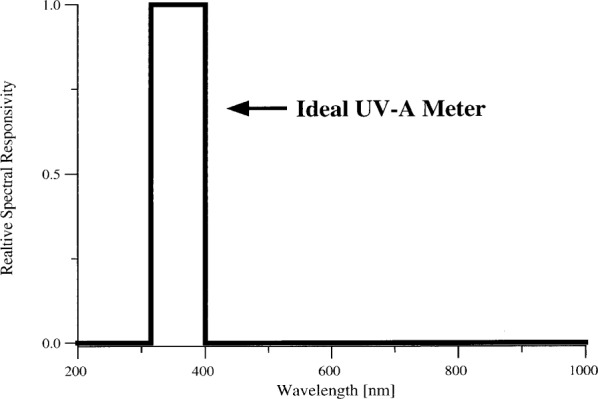
Spectral responsivity of an ideal UV-A meter.

An ideal meter would have a well-defined responsivity within a specific spectral region and zero responsivity outside of this region. For example, an ideal UV-A meter would have a constant responsivity from 315 nm to 400 nm and no response outside of this region. See Fig. 2.

**Fig. 3:**
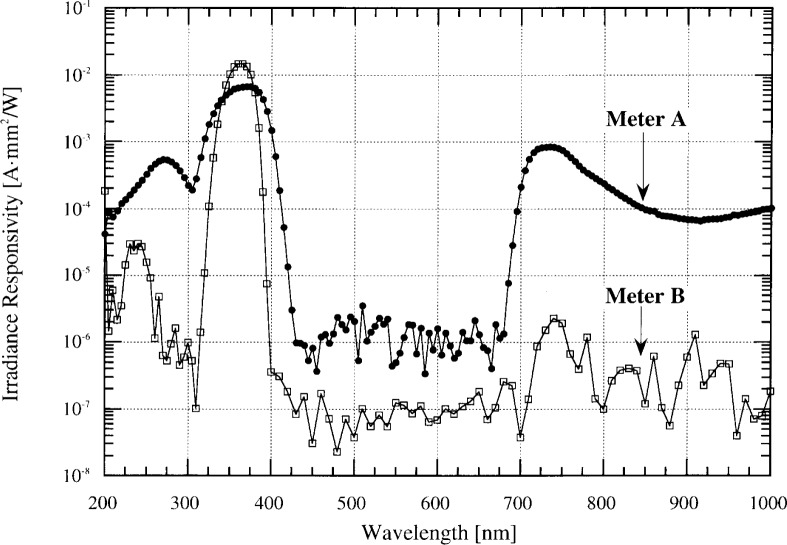
Spectral responsivity of two UV irradiance meters. Uncertainties for these data are given in Ref. [4].

Figure 3 shows the spectral responsivity, determined in monochromatic radiation, of two broadband UV meters used in semiconductor photolithography to determine the total exposure of a photoresist to 365 nm radiation from a filtered mercury source [4]. These meters have a maximum responsivity in the 365 nm region, and the responsivity then decreases to a much smaller, though non-zero, value at longer wavelengths. The instruments demonstrate differing amounts of increased responsivity in the near infrared (IR), with Meter A showing responsivity 2 to 3 orders of magnitude larger than Meter B in the 700 nm to 1000 nm spectral region. The increased IR responsivity is due to increased transmission in the IR by the glass filters, and because silicon photodiodes have their peak response in the near IR. The increased responsivity observed at wavelengths shorter than 300 nm is caused by fluorescence of the diffuser, which then re-emits longer wavelength radiation that passes through the filter to the photodiode. This was verified in Meter A by placing the diffuser between the filter and the photodiode. This effectively eliminated the responsivity near 275 nm.

For monochromatic radiation measurements near 365 nm, the out-of-band response is not important and both meters can make measurements with little error. Many real optical sources that are assumed monochromatic, such as lasers, often emit radiation at additional wavelengths. If the source to be measured emits flux at wavelengths below 300 nm or above 680 nm, the 365 nm radiation could be overestimated and measurements with these two meters will disagree.

**Fig. 4:**
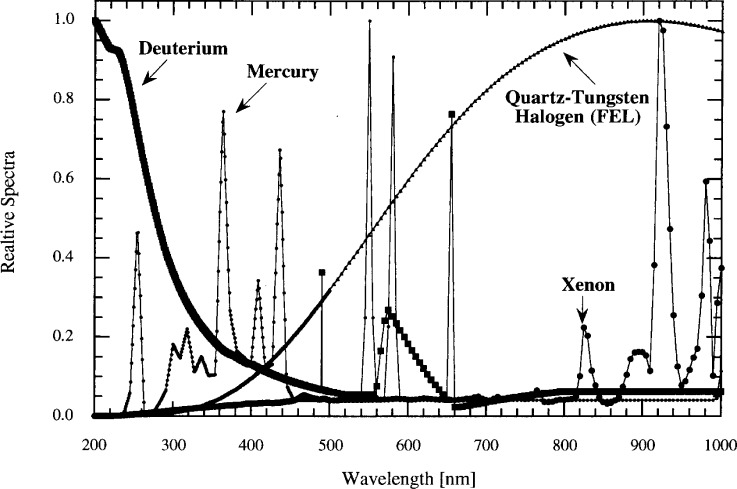
Typical source spectra used for comparison.

Although these UV meters were designed to measure monochromatic radiation, they are very similar to UV meters designed and used for broadband UV radiation. To illustrate these errors, we compare the signal produced by the two UV meters from four typical sources with different spectral power distributions: a mercury arc lamp, a quartz-tungsten halogen lamp (ANSI designation, FEL), a deuterium lamp, and a xenon arc lamp. The relative spectral distribution of each source is shown in Fig. 4.

Using Eq. (1), we compare the integrated in-band irradiance signal with the out-of-band signal. The in-band signal is the product of the spectral distribution of the source and the meter responsivity, integrated over the spectral region from 315 nm to 400 nm. The out-of-band response is the integral of the product summed over the 200 nm to 315 nm and 400 nm to 1000 nm spectral ranges.

The ratio of the out-of-band signal to the in-band signal for the two meters is shown in Table 1. There is a dramatic difference in the performance of the two instruments. The out-of-band contribution to the total signal from Meter B is at most 1.9 % for the deuterium source and 0 % for the Hg source. In contrast, the signal from Meter A is dominated by stray light when measuring an FEL lamp, with the out-of-band signal three and a half times larger than the in-band signal.

Similarly, calibrating with one type of source and subsequently measuring a different type can lead to large and undefined errors, as shown in Table 2 and Table 3. These errors shown are the relative differences in the ratio of the total signal from the UV meter for each source compared to the ratio of the actual UV-A in each source.

$$\text{Error}=\frac{S-E}{E},$$ (2)

where *S* and *E* are the ratios of the test over calibration source for the total signal measured and the actual UV-A light from the sources, respectively.

Note that if the calibration and test sources are identical, the error is zero. This is usually not possible to do in practice, and most users prefer to measure several types of sources with a UV meter. Note also that in Table 3 the errors are also large, even though there is low out-of-band response for this meter. This is because the in-band response does not match the ideal UV-A response function well.

## 4. Cosine Response

**Fig. 5:**
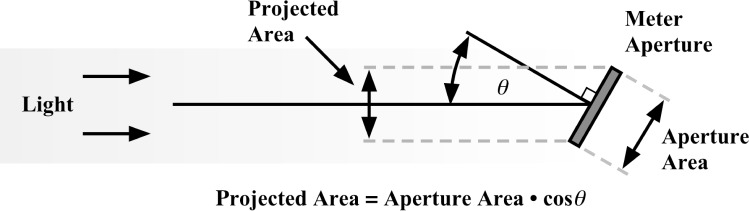
Geometry illustrating how angle effects the signal measured by a meter.

If a narrow beam of light, overfilling the aperture of a UV meter, is incident at an angle *θ* (where *θ* is the angle between the beam and the direction normal to the meter surface), the signal will be less than that for a beam perpendicular to the meter surface by the factor cos(*θ*), which is the ratio of the projected area of the meter aperture in the direction of the light beam to the absolute aperture area. See Fig. 5.

For an ideal meter, the spectral responsivity can be calibrated using an incident beam of any solid angle, and the meter can then be used to correctly measure light entering the meter aperture over any angular distribution. In reality, meters are not ideal and have a responsivity that decreases with angle faster than the cosine function. This is due to reflection or geometrical losses for light incident at larger angles. This non-ideal cosine response can lead to large errors if the calibration numerical aperture is substantially different from the measurement application.

**Fig. 6:**
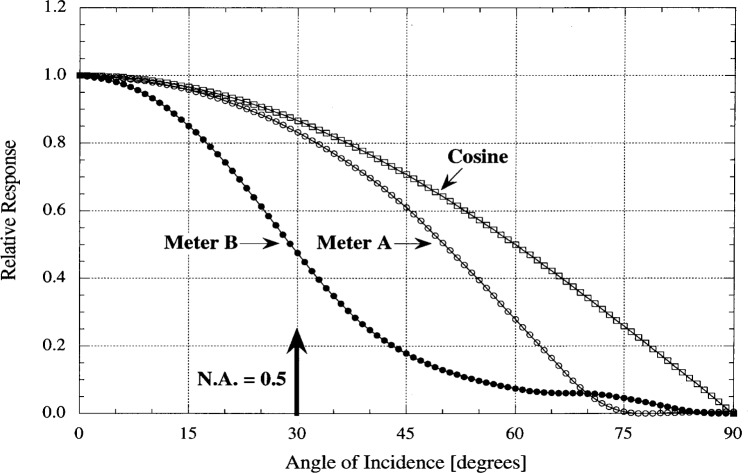
Angular response of two UV meters.

Each meter was tested by precisely rotating the meter about an axis at the aperture while irradiating the meter with a narrow light beam. The results of these measurements are shown in Fig. 6 [4]. From the figure, we see for Meter A responsivity to light at 30° from normal was 3.5 % less than ideal, and Meter B was low by 45 %. An angle of 30° is at the edge of a solid angle corresponding to a numerical aperture (NA) of 0.5. Commercial photolithography instruments often use such a large solid angle. Since an accurate determination of the spatial characteristics of the incoming light in a stepper is not feasible, the use of a meter with a large nearly ideal angular response is strongly recommended. Otherwise, error is introduced when a different incident irradiation geometry from the source being measured is used in the calibration of a meter.

**Fig. 7:**
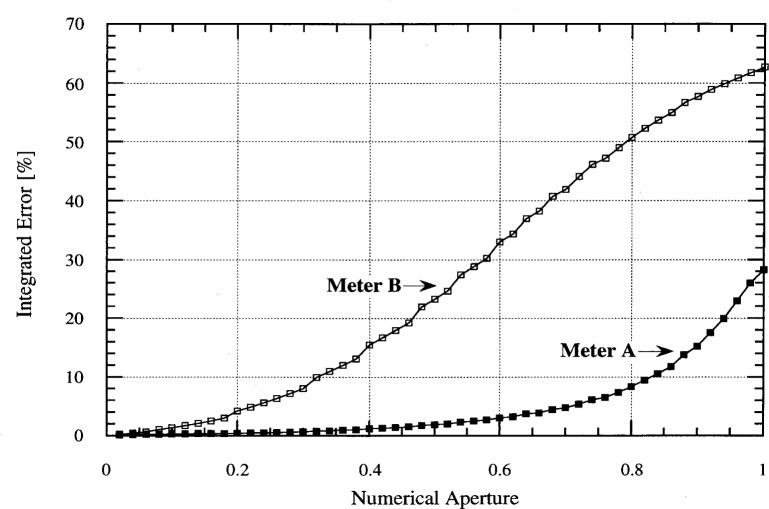
Integrated error due to cosine response.

If we assume that the irradiation from a particular source is uniform over the solid angle containing the radiation, we can calculate the difference between the response of an ideal meter and the ones presented in Fig. 6. This is shown in Fig. 7 as a function of numerical aperture [4]. For a NA of 0.5, for example, Meter A’s response would be too low by 2 % and Meter B’s by 23 %. Thus, a large deviation from cosine response leads to greater uncertainty in irradiance determinations. However, for small NA both meters can make measurements with little error.

In summary, Meter A had poorer out-of-band response than Meter B, but superior cosine response. Depending on the application, either meter may be the best choice.

## 5. Action Spectra

There have been successful implementations of broadband radiometers for the measurement of broadband sources. In many cases, these instruments have filters that shape the detector responsivity to approximate a particular spectral response function (action spectrum) for a specific application. An action spectrum is the effectiveness of different wavelengths of light to cause a photobiological or photochemical action or reaction. The most common example is a photopic detector, which is designed to approximate the human visual response.

**Fig. 8:**
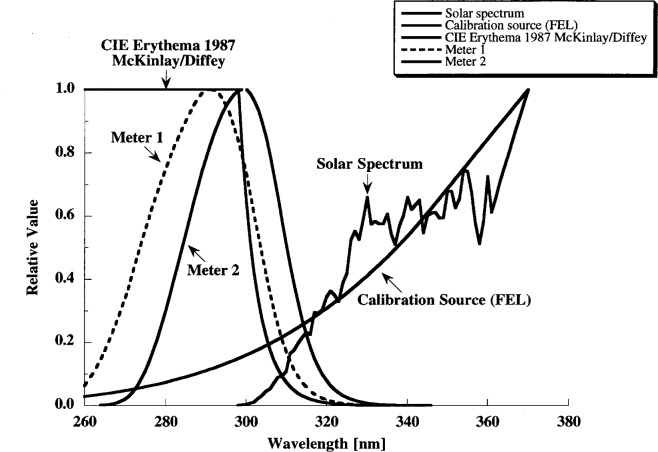
Spectral responsivities of two UV radiometers plotted with the CIE 1987 erythema function and spectral power distribution of an FEL lamp and the solar spectrum. (Fig. 3 in Ref. [6].)

In the UV spectral region, Roberson-Burger type meters have been developed for the measurement of erythemal (sunburn) effectiveness of sunlight. The relative spectral responsivities of two UV radiometers together with the CIE 1987 erythema function [5] are shown in Fig. 8 [6]. Note the large mismatch between the spectral responsivities of the two UV meters and the erythema function.

The spectral mismatch correction technique commonly used in photometry can be applied to UV broadband measurements where the photopic *V*(*λ*) function is replaced by a particular UV action spectrum. In this case, the erythema function replaces *V*(*λ*), and the spectral mismatch correction factors were calculated in Ref. 6 to be 0.48 for Meter 1 and 0.19 for Meter 2. The term $f_{1}^{\prime }$ is an evaluation index used to rate the spectral mismatch quality of photometers, and is not used for correction purposes. The $f_{1}^{\prime }$ values (given in Ref. 6) of the two radiometers for the erythema function are 38 % and 76 %, respectively. In contrast, photometers commonly have errors and $f_{1}^{\prime }$ values of a few percent, reflecting the more advanced photopic filter designs.

This example shows that large uncertainties result from calibrating these meters with FEL lamps, and then using the meter for measuring solar radiation or UV lamps. However, the errors will be small if both the calibration source and source to be measured are accurately known, and the spectral mismatch function is applied to the measurement result. If the calibration source is different from the source to be measured, correction factors are needed for each type of source to be measured. In addition, unlike in photometry, in UV measurements very few action spectra are well defined.

## 6. Conclusions

The amount of effort required in the calibration of an instrument and the care involved in its use depend on the particular measurement application and the desired uncertainty. For UV measurements with the lowest uncertainties, it is necessary to analyze the measurement problem; match the radiometer to the application; match the calibration source to the application measurement; and characterize the radiometer for its spectral and geometric response and any other parameter that will affect the measurement.

If the application involves measuring a monochromatic source, a simple broadband UV meter correctly calibrated at that wavelength is generally sufficient. If the measurements are of a broadband or extended source, it is best to pick a UV meter that has the closest match to the desired measurement function (such as UV-A or erythema). If that is not possible, then a correction factor can be calculated for each source to be measured, but one must have measured the absolute spectral responsivity of the UV meter and know the source’s relative spectral distribution. Alternately, one can calibrate the meter with a calibration source that is similar in its spectral distribution to the source being measured.

## Figures and Tables

**Table 1:** Ratio of the out-of-band to in-band signal for different sources

Ratio (out-of band/in-band)		Source measured		
	FEL	Mercury	Deuterium	Xenon
Meter A	363.8 %	7.4 %	35.2 %	68.1 %
Meter B	0.8 %	0.0 %	1.9 %	0.2 %

**Table 2:** Expected error when measuring differing test and calibration sources for Meter A

Calibration source		Test source		
	FEL	Mercury	Deuterium	Xenon
FEL	0.0 %	−74.8 %	−74.7 %	−64.9 %
Mercury	297.0 %	0.0 %	0.3 %	39.5 %
Deuterium	296.0 %	−0.3 %	0.0 %	39.2 %
Xenon	184.6 %	−28.3 %	−28.1 %	0.0 %

**Table 3:** Expected Error when measuring differing test and calibration sources for Meter B

Calibration source		Test source		
	FEL	Mercury	Deuterium	Xenon
FEL	0.0 %	50.8 %	−6.7 %	2.7 %
Mercury	−33.7 %	0.0 %	−38.2 %	−31.9 %
Deuterium	7.2 %	61.7 %	0.0 %	10.1 %
Xenon	−2.7 %	46.8 %	−9.2 %	0.0 %
